# Preoperative neutrophil-to-lymphocyte ratio correlates with PD-L1 expression in immune cells of patients with malignant pleural mesothelioma and predicts prognosis

**DOI:** 10.1038/s41598-023-31448-4

**Published:** 2023-03-31

**Authors:** Riki Okita, Nobutaka Kawamoto, Masanori Okada, Hidetoshi Inokawa, Naoki Yamamoto, Tomoyuki Murakami, Eiji Ikeda

**Affiliations:** 1 Department of Thoracic Surgery, National Hospital Organization Yamaguchi Ube Medical Center, 685 Higashikiwa, Ube Yamaguchi, 755-0241 Japan; 2 Health Administration Center, Yamaguchi University Graduate School of Medicine, 1-1-1 Minami-Kogushi, Ube, Yamaguchi, 755-8505 Japan; 3 Department Clinical Research, National Hospital Organization Yamaguchi Ube Medical Center, 685 Higashikiwa, Ube Yamaguchi, 755-0241 Japan; 4 Department of Pathology, KYURIN/ KYURIN PACELL Corporation, 26-67 Morishita-Cho, Yahatanishi-Ku, Kitakyushu, Fukuoka 806-0046 Japan; 5 Department of Pathology, Yamaguchi University Graduate School of Medicine, 1-1-1 Minami-Kogushi, Ube, Yamaguchi, 755-8505 Japan

**Keywords:** Cancer, Immunology, Oncology, Risk factors

## Abstract

We assessed the prognostic value of five complex inflammatory and nutritional parameters, namely neutrophil-to-lymphocyte ratio (NLR), prognostic nutritional index (PNI), C-reactive protein-to-NLR ratio (C/NLR), platelet-to-lymphocyte ratio (PLR), and lymphocyte-to-monocyte ratio (LMR) using data from patients with malignant pleural mesothelioma (MPM) undergoing extrapleural pneumonectomy (EPP). Moreover, the correlation between these five parameters and programmed cell death protein 1 ligand-1 (PD-L1) expression in the tumor microenvironment was evaluated. This study included consecutive MPM patients who underwent EPP. The histological subtype of the eligible patients (n = 61) correlated with all five parameters. Moreover, the PD-L1 expression scores for immune cells correlated with NLR and PLR, and the PD-L1 expression scores for both tumor cells and immune cells were inversely correlated with both PNI and LMR. Univariate analysis elucidated that NLR, PNI, and C/NLR were predictors of 5-year overall survival (OS), and multivariate analysis revealed that NLR was an independent predictor of 5-year OS, suggesting that NLR is a preoperative, prognostic factor for patients with MPM who are scheduled for EPP. To the best of our knowledge, this is the first study to evaluate the prognostic potentials of NLR, PNI, C/NLR, PLR, and LMR simultaneously in patients with MPM who underwent EPP.

## Introduction

Malignant pleural mesothelioma (MPM) is one of the most aggressive solid tumors, although multimodal therapies^1^, as well as immune checkpoint inhibitors targeting the programmed cell death protein-1 (PD1)/programmed cell death protein-1 ligand 1 (PD-L1) axis^2,3^ have improved the clinical outcome of patients with MPM. Recently, several preoperative inflammatory and nutritional parameters such as neutrophil-to-lymphocyte ratio (NLR), prognostic nutritional index (PNI), and C-reactive protein (CRP)-to-albumin ratio have been reported as prognostic factors for surgical treatment of several types of malignant tumor^4–6^. Inflammatory parameters are useful for the prognosis of MPM, as asbestos-induced chronic inflammation is associated with the development of MPM^7^. We previously showed that the CRP-to-albumin ratio and CRP multiplied by the neutrophil ratio predicted the prognosis of patients with MPM undergoing extrapleural pneumonectomy (EPP)^8^, while most studies that have investigated NLR and PNI in MPM included inoperable cases^7,9–11^. Thus, the relationship between inflammatory and nutritional parameters and surgical outcomes in patients with MPM remains unknown.

PD-L1 is a well-known immune checkpoint molecule that suppresses T cell-induced anti-tumor immunity^12^. Previous studies have reported that in patients with MPM, PD-L1 overexpression in the tumor microenvironment (TME) is a poor prognostic factor^13–15^, but other studies, including our previous study, revealed that PD-L1 status has no influence on the prognosis of patients with MPM^16–18^. Although the prognostic impact of PD-L1 status in patients with MPM is controversial, the expression status of PD-L1 can be used as a preoperative prognostic marker because it can be determined via pleural biopsy. Furthermore, the relationship between PD-L1 expression and inflammatory parameters is interesting, since inflammatory cytokines directly induce PD-L1 expression in tumor cells^19,20^. Moreover, the relationship between PD-L1 and nutritional parameters is another important criteria because changes in nutritional status impact immune cell metabolism and function^21^; undernutrition is associated with immunosuppression, which manifests via a decrease in the immune cell number, particularly that of T cells^22^.

In this study, preoperative laboratory biomarkers for systemic inflammation and nutrition, namely NLR, PNI, CRP to NLR ratio (C/NLR), platelet-to-lymphocyte ratio (PLR), and lymphocyte-to-monocyte ratio (LMR), were evaluated, and we determined the relationship between each inflammatory or nutritional parameter and PD-L1 expression in the TME. Moreover, the prognostic impact of the five inflammatory/nutritional parameters was evaluated in patients with MPM who underwent EPP.

## Materials and methods

### Patients and specimens

This retrospective, single-center, observational study was approved by the Ethics Committee of the National Hospital Organization Yamaguchi Ube Medical Center (No. 30-5) with following consent style; written informed consent for the use of medical records and tissue samples was obtained from all living patients or patients treated at our hospital after October 2012, while written informed consent was waived; an opt-out method on the website of the National Hospital Organization Yamaguchi Ube Medical Center was applied to obtain patient consent if the patients who treated before October 2012 were dead before April 30, 2021. The study adheres to the principles of the Declaration of Helsinki. Consecutive patients who underwent EPP for MPM and achieved macroscopic complete resection (MCR) between June 2006 and March 2020 were enrolled in this study. Our routine treatment strategy for operable MPM, the method for patient follow-up, and data regarding this patient population have been described in our previous study^8^. Pleurodesis was not routinely performed after pleural biopsy. The interval between biopsy and blood test results before EPP is usually 3–4 weeks because it takes 3 weeks for a pathological diagnosis by immunohistochemistry (IHC) of pleural biopsy specimens. If patients had undergone pleurodesis at another hospital, EPP was performed after inflammation returned to normal or the level before pleurodesis, to minimize the disadvantages of pleurodesis-induced inflammation in the perioperative management of EPP. Patients with empyema were excluded from surgical indication, because EPP is a highly invasive procedure that requires the reconstruction of the diaphragm and pericardium with polytetrafluoroethylene patches. Five-year overall survival (OS) was estimated from the date of EPP to the date of death from any cause or the last follow-up, which was set to a maximum of 5 years, and the last follow-up date was April 30, 2021. Only OS (with a limited observation period of ≤ 5 years) was evaluated because the majority of patients were followed up at other hospitals, making it difficult to collect accurate information on cause of death and long-term prognosis. Histological diagnosis was according to the WHO 2015 criteria^23^. TNM classification and pathological stage (pStage) were assessed as per the criteria defined by the International Association for the Study of Lung Cancer^24–26^.

### Clinical variables

The following clinicopathological data of the patients were obtained for the analysis: age, sex, laterality, histological subtype, and pathological stage. Moreover, laboratory blood test results, including white blood cell count (WBC), platelet count, neutrophil count, lymphocyte count, monocyte count, serum CRP level, and serum albumin level, were obtained; these tests were performed within 7 days before EPP. Preoperative inflammatory and nutritional parameters were calculated as follows: NLR, neutrophil count divided by lymphocyte count; PNI, 10 × serum albumin + 0.005 × lymphocyte count^27^; C/NLR, serum CRP level divided by NLR; PLR, platelet count divided by lymphocyte count; and LMR, lymphocyte count divided by monocyte count.

### IHC

Formalin-fixed paraffin-embedded blocks from 61 MPM patients were obtained from the archives of the National Hospital Organization Yamaguchi Ube Medical Center. IHC was performed on surgically resected tissues using a mouse monoclonal anti-PD-L1 antibody (1:100, clone SP142, Spring Bioscience, Pleasanton, CA, USA), according to a previously described protocol^18^. The scoring criteria for PD-L1 expression by tumor cells (PD-L1 TC) and immune cells (PD-L1 IC) were used to assess the percentage of total tumor and immune cells, respectively, as per a previously described method^18,28^. Immunoreactivity for PD-L1 was scored by two investigators (R.O. and N.K. or N.Y.), who had no prior knowledge of the corresponding clinicopathological data. To evaluate the status of PD-L1 TC and PD-L1 IC, average scores were calculated from the two investigators.

### Statistical analysis

The cutoff values of each parameter were determined according to the receiver operating characteristic (ROC) curves predicting 5-year OS, using the SPSS statistical package (version 17.0; SPSS, Chicago, IL). Chi-square test was performed to evaluate the relationships between patient characteristics and each parameter, and Spearman’s rank correlation test was performed for correlation analysis using GraphPad Prism (version 6.01; GraphPad Software, La Jolla, CA). The 5-year OS rate until death or last follow-up after EPP was evaluated by Kaplan–Meier survival analysis, and the significance of the differences in 5-year OS between groups was assessed by the log-rank Mantel–Cox test using GraphPad Prism. Univariate and multivariate analyses were performed using the Cox proportional hazards model to identify independent prognostic factors, using SPSS statistical package 17.0. The statistical significance was set at* p* < 0.05.

### Ethics approval

This research was approved by the Yamaguchi Ube Medical Center ethics committee (No. 30-5); the study followed the principles of the Declaration of Helsinki and was in compliance with the relevant guidelines and regulations.

### Consent to participate

Written informed consent for the use of tissue samples and medical records was waived, and an opt-out method on the website was applied to obtain patient consent; however, written informed consent was obtained from all living patients or patients treated in our hospital after October 2012.

## Results

### Patient cohort

We previously assessed PD-L1 TC and IC based on representative IHC results in 58 EPP cases of MPM to evaluate their prognostic impact^18^. In the present study, the number of patients was higher and the relationship between inflammatory and nutritional parameters and the expression status of PD-L1 in the TME of MPM was determined. This study included 61 consecutive patients with MPM who underwent EPP and achieved MCR. The patient characteristics are described previously, and the median length of follow-up for censored cases was 39.7 months (range, 20.2–59.3 months)^8^.

### Preoperative inflammatory and nutritional parameters of patients with resected MPM

The WBC, platelet count, serum CRP level, serum albumin level, NLR, PNI, C/NLR, PLR, and LMR of the patients were 3180–10,700 cells/μL (mean: 6233), 11.2–53.2 × 10^4^ cells/μL (mean 28.1), 0.01–10.48 mg/dL (mean: 1.24), 2.4–4.8 g/dL (mean: 3.7), 1.1–5.7 (mean: 2.5), 29.9–64.5 (mean: 45.1), 0.01–32.58 (mean: 4.20), 62.2–436.3 (mean: 184.9), and 1.2–10.3 (mean: 4.5), respectively. PD-L1 TC score was 0–3 (mean: 1.30) and PD-L1 IC score was 0–3 (mean: 1.45). Optimal cutoff values for each parameter determined by ROC analyses were as follows: 5965 cells/μL for WBC, 28.35 × 10^4^ cells/μL for platelet count, 1.75 for PD-L1 TC score, 1.75 for PD-L1 IC score, 2.850 for NLR, 45.150 for PNI, 0.955 for C/NLR, 167.1 for PLR, and 5.15 for LMR (Supplementary Fig. 1, 2). Optimal cutoff values for CRP and albumin levels were 0.515 mg/dL and 3.75 g/dL, respectively, as proposed previously^8^. Next, the patients were divided into high and low groups based on the optimal cut-off value of each parameter for further analysis. The inflammatory and nutritional parameters of the patients are presented in Table 1.

**Table 1:** Inflammatory or nutritional parameters characteristics.

Characteristics cutoff value	Mean (range) number of the case
WBC	6233 (3180–10,700)
< 5965	30
5965 ≦	31
Plt	28.1 (11.2–53.2)
< 28.35	34
28.35 ≦	27
CRP	1.24 (0.01–10.48)
< 0.515	37
0.515 ≦	24
Albumin	3.7 (2.4–4.8)
< 3.750	32
3.750 ≦	29
NLR	2.5 (1.1–5.7)
< 2.850	42
2.850 ≦	19
PNI	45.1 (29.9–64.5)
< 45.150	31
45.150 ≦	30
C/NLR	4.20 (0.01–32.58)
< 0.955	34
0.955 ≦	27
PLR	184.9 (62.2–436.3)
< 167.1	30
167.1 ≦	31
LMR	4.5 (1.2–10.3)
< 5.15	39
5.15 ≦	22
PD-L1 TC	1.30 (0–3)
< 1.75	39
1.75 ≦	22
PD-L1 IC	1.45 (0–3)
< 1.75	27
1.75 ≦	34

*WBC* white blood cell count, *Plt* platelet count, *CRP* C-reactive protein, *NLR* neutrophil to lymphocyte ratio, *PNI* prognostic nutritional index, *C/NLR* CRP divide by NLR, *PLR* platelet to lymphocyte ratio, *LMR* lymphocyte to monocyte ratio, *PD-L1 TC* PD-L1 by tumor cells, *PD-L1 IC* PD-L1 by immune cells.

### Relationship between clinicopathological characteristics and the inflammatory and nutritional parameters

**Figure 1:**
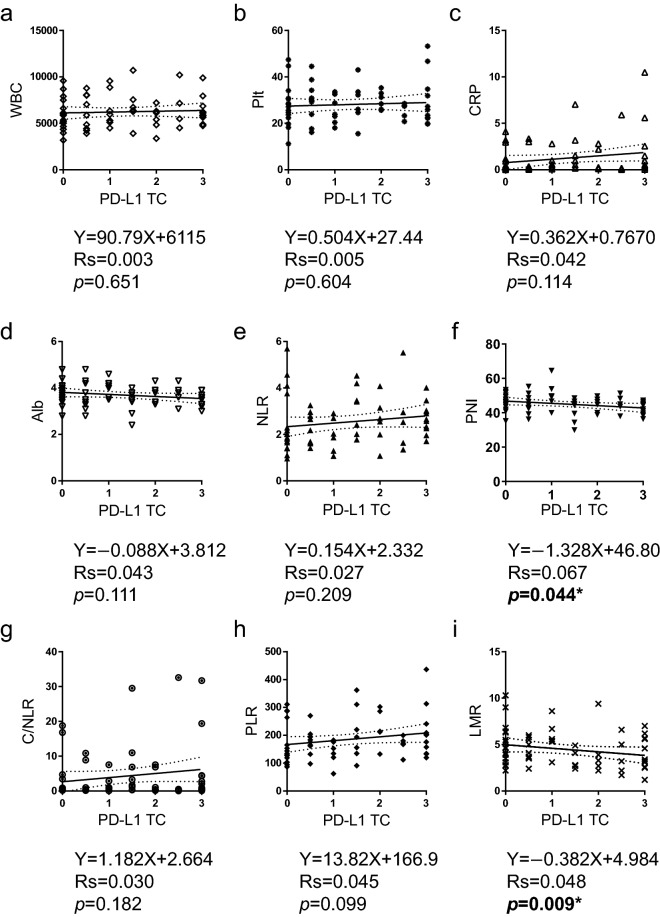
PD-L1 TC inversely correlates with PNI and LMR. Correlations between PD-L1 TC and (**a**) WBC, (**b**) platelet count, (**c**) CRP level, (**d**) Alb level, (**e**) NLR, (**f**) PNI, (**g**) C/NLR, (**h**) PLR, or (**i**) LMR. Data were analyzed using the Spearman’s rank correlation test. PD-L1 TC, programmed cell death 1 ligand-1 by tumor cells; WBC, white blood cell count; Plt, platelet count; CRP, C-reactive protein; Alb, albumin; NLR, neutrophil-to-lymphocyte ratio; PNI, prognostic nutritional index; C/NLR, C-reactive protein-to-NLR ratio; PLR, platelet-to-lymphocyte ratio; LMR, lymphocyte-to-monocyte ratio; Rs, Spearman’s rank correlation coefficient. *p < 0.05.

**Figure 2:**
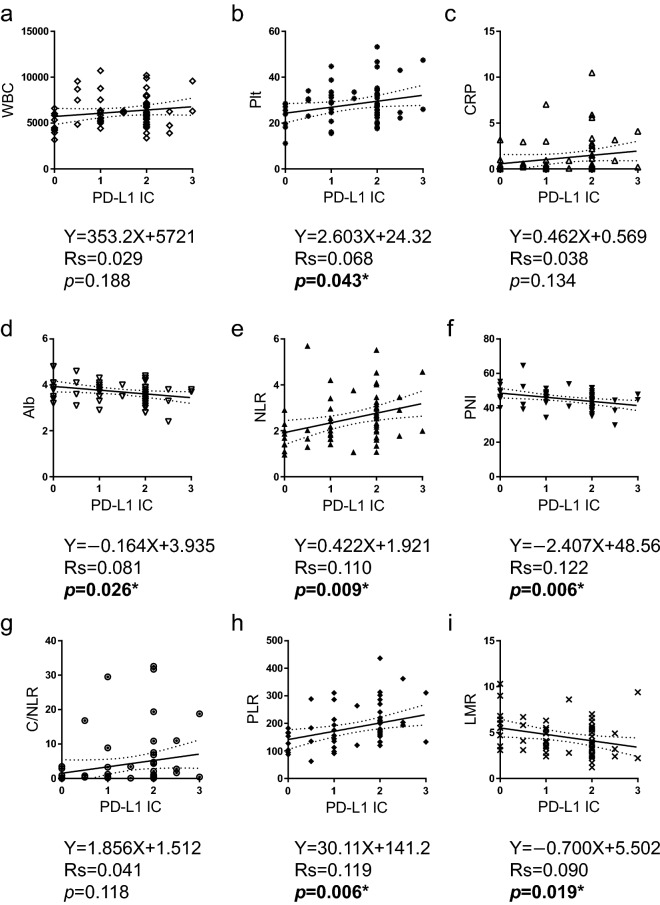
PD-L1 IC positively correlates with platelet count, NLR, and PLR, whereas inversely correlates with albumin, PNI and LMR. Correlation between PD-L1 IC and (**a**) WBC, (**b**) platelet count, (**c**) CRP level, (**d**) Alb level, (**e**) NLR, (**f**) PNI, (**g**) C/NLR, (**h**) PLR, and (**i**) LMR. Data were analyzed using the Spearman’s rank correlation test. PD-L1 IC: programmed cell death 1 ligand-1 by immune cells; WBC: white blood cell count; Plt: platelet count; CRP: C-reactive protein; Alb: albumin; NLR: neutrophil-to-lymphocyte ratio; PNI: prognostic nutritional index; C/NLR: C-reactive protein-to-NLR ratio; PLR: platelet-to-lymphocyte ratio; LMR: lymphocyte-to-monocyte ratio; Rs: Spearman’s rank correlation coefficient. *p < 0.05.

**Figure 3:**
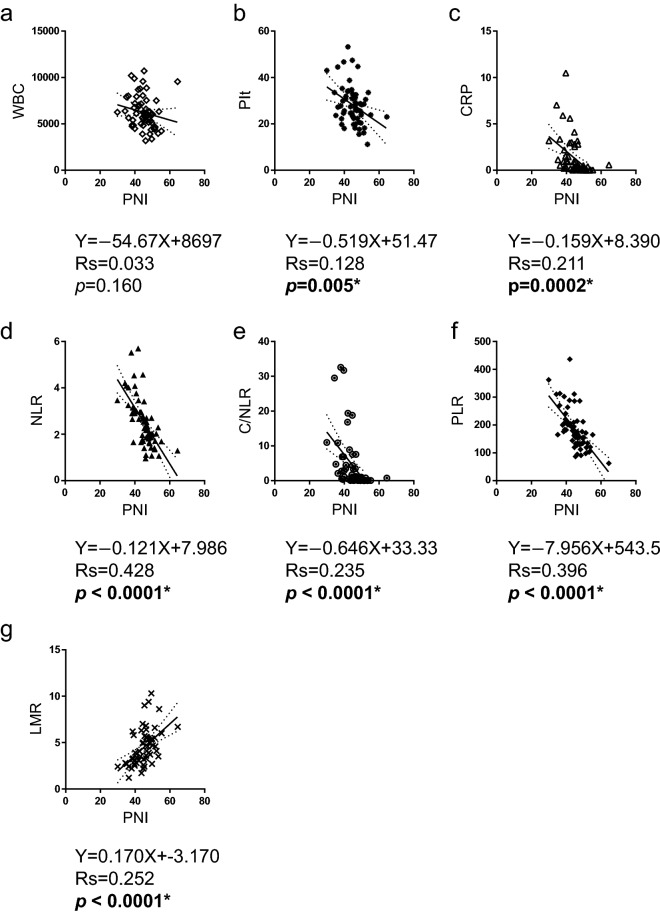
PNI positively correlates with LMR and inversely correlates with platelet count, CRP, NLR, C/NLR and PLR. Correlation between PNI and (**a**) WBC, (**b**) platelet count, (**c**) CRP level, (**d**) NLR, (**e**) C/NLR, (**f**) PLR, or (**g**) LMR. Data were analyzed using the Spearman’s rank correlation test. PNI, prognostic nutritional index; WBC, white blood cell count; Plt, platelet count; CRP, C-reactive protein; NLR, neutrophil-to-lymphocyte ratio; C/NLR, C-reactive protein-to-NLR ratio; PLR, platelet-to-lymphocyte ratio; LMR, lymphocyte-to-monocyte ratio; Rs, Spearman’s rank correlation coefficient. *p < 0.05.

The clinicopathological characteristics of the patients were compared according to the status of each inflammatory and nutritional parameter. Among the combination parameters, NLR was associated with histology, WBC, CRP, albumin, PNI, C/NLR, PLR, and LMR; PNI was associated with histology, CRP, albumin, PD-L1 IC, NLR, C/NLR, PLR, and LMR; C/NLR was associated with histology, WBC, CRP, albumin, PD-L1 IC, NLR, PNI, PLR, and LMR; PLR was associated with histology, platelet count, CRP, albumin, PD-L1 TC, NLR, PNI, C/NLR, and LMR; and LMR was associated with histology, WBC, CRP, NLR, PNI, C/NLR, and PLR (Table 2). The correlations between each parameter were evaluated to validate these results. Linear regression with Spearman’s rank correlation analysis revealed that the PD-L1 TC score was inversely correlated with PNI (Spearman’s rank correlation coefficient rs = 0.067, *p* = 0.044) and LMR (rs = 0.048, *p* = 0.009), although no correlation with other parameters was observed (Fig. 1). The PD-L1 IC score was positively correlated with platelet count (rs = 0.068, *p* = 0.043), NLR (rs = 0.110, *p* = 0.009), and PLR (rs = 0.119, *p* = 0.006), and inversely correlated with albumin level (rs = 0.081, *p* = 0.026), PNI (rs = 0.122, *p* = 0.006), and LMR (rs = 0.090, *p* = 0.019) (Fig. 2). PNI was inversely correlated with platelet count (rs = 0.128, *p* = 0.005), CRP (rs = 0.211, *p* = 0.0002), NLR (rs = 0.428, *p* < 0.0001), C/NLR (rs = 0.235, *p* < 0.0001), and PLR (rs = 0.396, *p* < 0.0001) and positively correlated with LMR (rs = 0.252, *p* < 0.0001) (Fig. 3).

**Table 2:** Clinicopathological characteristics by status of Inflammatory or nutritional parameters characteristics in 61 patients with MPM.

		NLR			PNI			C/NLR			PLR			LMR		
		Low	High	*p* value	Low	High	*p* value	Low	high	*p* value	Low	High	*p* value	Low	High	*p* value
Histology	Epi	37	7	< 0.001*	18	26	0.013*	30	14	0.002*	27	17	0.002*	24	20	0.014*
	Non-epi	5	12		13	4		4	13		3	14		15	2	
pStage	I-II	26	9	0.288	17	18	0.684	22	13	0.194	17	18	0.912	21	14	0.458
	III-IV	16	10		14	12		12	14		13	13		18	8	
WBC	Low	26	4	0.003*	12	18	0.096	23	7	0.001*	15	15	0.900	15	15	0.026*
	High	16	15		19	12		11	20		15	16		24	7	
Plt	Low	26	8	0.149	14	20	0.091	22	12	0.114	24	10	< 0.001*	19	15	0.142
	high	16	11		17	10		12	15		6	21		20	7	
CRP	Low	35	2	< 0.001*	10	27	< 0.001*	33	4	< 0.001*	24	13	0.002*	18	19	0.002*
	High	7	17		21	3		1	23		6	18		21	3	
Albumin	Low	13	19	< 0.001*	28	4	< 0.001*	8	24	< 0.001*	10	22	0.003*	24	8	0.059
	High	29	0		3	26		26	3		20	9		15	14	
PD-L1 TC	Low	29	10	0.216	17	22	0.133	22	17	0.888	24	15	0.010*	23	16	0.283
	High	13	9		14	8		12	10		6	16		16	6	
PD-L1 IC	low	21	6	0.180	9	18	0.015*	19	8	0.040*	17	10	0.055	15	12	0.225
	High	21	13		22	12		15	19		13	21		24	10	
NLR	Low	—			12	30	< 0.001*	33	9	< 0.001*	28	14	< 0.001*	20	22	< 0.001*
	High				19	0		1	18		2	17		19	0	
PNI	Low	12	19	< 0.001*	—			8	23	< 0.001*	6	25	< 0.001*	26	5	0.001*
	High	30	0					26	4		24	6		13	17	
C/NLR	Low	33	1	< 0.001*	8	26	< 0.001*	—			22	12	0.007*	15	19	< 0.001*
	High	9	18		23	4					8	19		24	3	
PLR	Low	28	2	< 0.001*	6	24	< 0.001*	22	8	0.007*	—			13	17	0.001*
	High	14	17		25	6		12	19					26	5	
LMR	Low	20	19	< 0.001*	26	13	0.001*	15	24	< 0.001*	13	26	0.001*	—		
	High	22	0		5	17		19	3		17	5				

*pStage* pathological stage, *WBC* white blood cell count, *Plt* platelet count, *CRP* C-reactive protein, *PD-L1 TC* PD-L1 by tumor cells, *PD-L1 IC* PD-L1 by immune cells, *NLR* neutrophil to lymphocyte ratio, *PNI* prognostic nutritional index, *C/NLR* CRP divide by NLR, *PLR* platelet to lymphocyte ratio, *LMR* lymphocyte to monocyte ratio.

*p < 0.05. Significant values are in bold.

### Five-year OS stratified by the inflammatory and nutritional parameters

**Figure 4:**
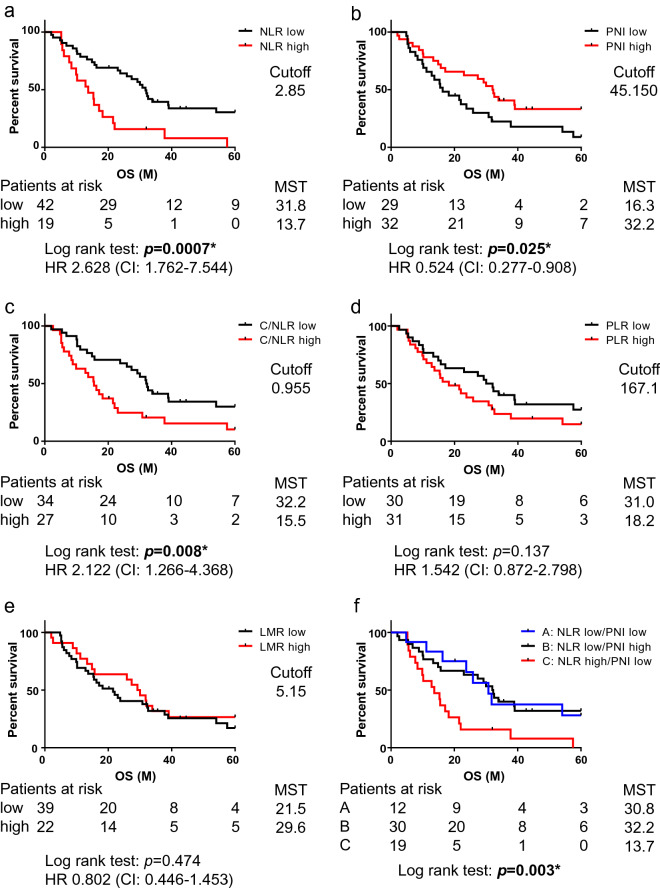
Association of a combination of inflammatory and nutritional parameters with OS in MPM patients who underwent EPP. Kaplan–Meier plots showing 5-year OS in patients with lower or higher scores for (**a**) NLR, (**b**) PNI, (**c**) C/NLR, (**d**) PLR, (**e**) LMR, and (**f**) combination of NLR and PNI. NLR, neutrophil-to-lymphocyte ratio; PNI, prognostic nutritional index; C/NLR, C-reactive protein-to-NLR ratio; PLR, platelet-to-lymphocyte ratio; LMR, lymphocyte-to-monocyte ratio; HR, hazard ratio; CI, confidence interval; MST, median survival time (months). *p < 0.05.

Kaplan–Meier survival analysis with log-rank test for 5-year OS demonstrated that the patients with high NLR (> 2.850) [hazard ratio (HR): 2.628, confidence interval (CI): 1.762–7.544, *p* = 0.0007] and high C/NLR (> 0.955) (HR: 2.122, CI: 1.266–4.368, *p* = 0.008) were significantly associated with poor prognosis, whereas those with high PNI (> 45.150) (HR: 0.524, CI: 0.277–0.908, *p* = 0.025) were significantly associated with good prognosis, and neither PLR status nor LMR status had an impact on the 5-year OS (Fig. 4a–e). For further analysis, the cases were divided into four groups according to high and low NLR (cutoff: 2.850), and high and low PNI (cutoff: 45.150), although no case of high NLR/high PNI was seen. We determined that the prognosis was similar between cases with low NLR with low PNI and low NLR with high PNI, whereas patients with high NLR with low PNI, which is the same as high NLR with any PNI, exhibited shorter survival compared to that of others (*p* = 0.003) (Fig. 4f).

### Univariate and multivariate analysis of the inflammatory and nutritional parameters for 5-year OS

Cox regression analysis was performed to determine the predictive value of the clinical variables for 5-year OS. For the analysis, NLR, PNI, C/NLR, PLR, and LMR were assessed independently with three other parameters, including sex, histological subtype, and laterality, as these parameters could be evaluated preoperatively. Both sex^29^ and histology^29,30^ are prognostic factors, and laterality is an important predictive factor for major morbidity^31^. Univariate analysis showed that sex (HR:3.231, *p* = 0.015), histological subtype (HR: 0.400, *p* = 0.004), NLR (HR: 2.790, *p* = 0.001), PNI (HR: 0.515, *p* = 0.027), and C/NLR (HR: 2.183, *p* = 0.009) were significantly associated with 5-year OS. Multivariate analyses revealed that NLR was an independent predictor for 5-year OS in the NLR-including cohort (HR 2.062, CI: 1.054–4.031, *p* = 0.034), whereas PNI, C/NLR, PLR and LMR exhibited no significant impact on 5-year OS in each cohort, although histology was an independent prognostic factor in these four cohorts and sex was an independent prognostic factor in the cohorts with PLR or LMR (Table 3).

**Table 3:** Cox proportional hazard model for 5-year OS (n = 61).

	Univariate		Multivariate	
	Hazard ratio (95% CI)	*p* value	Hazard ratio (95% CI)	*p* value
Sex (male vs female)	3.231 (1.259–8.290)	0.015*	2.566 (0.981–6.711)	0.055
Latelarity (right vs left)	0.902 (0.504–1.613)	0.728	0.652 (0.346–1.227)	0.185
Histology (epi. vs non-epi)	0.400 (0.215–0.742)	0.004*	0.518 (0.257–1.044)	0.066
NLR (low vs high)	2.790 (1.509–5.156)	0.001*	2.062 (1.054–4.031)	0.034*

	Univariate		Multivariate	
	Hazard ratio (95% CI)	*p* value	Hazard ratio (95% CI)	*p* value
Sex (male vs female)	3.231 (1.259–8.290)	0.015*	2.583 (0.990–6.740)	0.052
Latelarity (right vs left)	0.902 (0.504–1.613)	0.728	0.687 (0.368–1.281)	0.237
Histology (epi. vs non-epi)	0.400 (0.215–0.742)	0.004*	0.427 (0.219–0.832)	0.012*
PNI (low vs high)	0.515 (0.286–0.928)	0.027*	0.619 (0.341–1.124)	0.115

	Univariate		Multivariate	
	Hazard ratio (95% CI)	*p* value	Hazard ratio (95% CI)	*p* value
Sex (male vs female)	3.231 (1.259–8.290)	0.015*	2.600 (0.999–6.771)	0.050
Latelarity (right vs left)	0.902 (0.504–1.613)	0.728	0.685 (0.366–1.280)	0.235
Histology (epi. vs non-epi)	0.400 (0.215–0.742)	0.004*	0.457 (0.232–0.899)	0.023*
C/NLR (low vs high)	2.183 (1.211–3.937)	0.009*	1.816 (0.993–3.321)	0.053

	Univariate		Multivariate	
	Hazard ratio (95% CI)	*p* value	Hazard ratio (95% CI)	*p* value
Sex (male vs female)	3.231 (1.259–8.290)	0.015*	2.782 (1.055–7.340)	0.039*
Latelarity (right vs left)	0.902 (0.504–1.613)	0.728	0.692 (0.372–1.290)	0.247
Histology (epi. vs non-epi)	0.400 (0.215–0.742)	0.004*	0.421 (0.204–0.869)	0.019*
PLR (low vs high)	1.553 (0.865–2.788)	0.140	1.021 (0.518–2.012)	0.952

	Univariate		Multivariate	
	Hazard ratio (95% CI)	*p* value	Hazard ratio (95% CI)	*p* value
Sex (male vs female)	3.231 (1.259–8.290)	0.015*	2.778 (1.061–7.278)	0.038*
Latelarity (right vs left)	0.902 (0.504–1.613)	0.728	0.695 (0.374–1.289)	0.248
Histology (epi. vs non-epi)	0.400 (0.215–0.742)	0.004*	0.419 (0.217–0.808)	0.010*
LMR (low vs high)	0.801 (0.436–1.472)	0.475	0.963 (0.520–1.784)	0.904

*OS* overall survival, *CI* confidence interval, *epi* epithelioid type, *NLR* neutrophil to lymphocyte ratio, *PNI* prognostic nutritional index, *C/NLR* CRP divide by NLR, *PLR* platelet to lymphocyte ratio, *LMR* lymphocyte to monocyte ratio.

* *p* < 0.05. Significant values are in bold.

## Discussion

In this study, we elucidated that NLR is an independent prognostic factor for the outcome of EPP in patients with MPM. Moreover, we found that PD-L1 TC score was inversely correlated with PNI and LMR; PD-L1 IC score was positively correlated with platelet count, NLR, and PLR and inversely correlated with albumin level, PNI, and LMR.

Elevated neutrophil and platelet counts are associated with systemic inflammation. Neutrophils promote transformation, progression, and metastasis of cancer cells while suppressing antitumor immunoreaction by proinflammatory cytokines^32^. Platelet-tumor interaction is important because thrombocytosis is associated with shorter survival in patients with unresectable MPM^33^. During thrombopoiesis, the mobilization of hematopoietic stem cells is controlled by endothelial-active cytokines, such as vascular endothelial growth factor^34^, which are upregulated during pleural effusion in patients with MPM^35^; they are secreted directly by tumor cells and indirectly by tumor-infiltrating immune cells due to tumor-related inflammation^36^.

The relationship between the five parameters investigated in this study and PD-L1 expression is relevant because both inflammation and nutrition affect immunological functions. Regarding LMR, monocytes release several immunosuppressive cytokines and create oxidative stress, which promotes tumor progression^37^. One important immunosuppressive phenotype of monocytes is myeloid-derived suppressor cells (MDSC), and their count increases in the TME of patients with MPM^38^. In an experimental mouse model, the tumor inflammatory microenvironment supported MDSC recruitment, and tumor-derived MDSC inactivated T cell proliferation^39^. Moreover, in a tumor-bearing mouse model, the inhibition of PD-L1 decreased MDSC count and increased effector-memory T cell count^40^. In the present study, LMR had no impact on clinical outcomes, although tumors expressing PD-L1 might have an increased number of circulating monocytes, including MDSC, whereas a decreased number of circulating effector-memory lymphocytes, which play a pivotal role in eliminating tumor cells, as LMR is inversely correlated with PD-L1 TC and IC. One reason behind why PD-L1 TC score was inversely correlated with PNI could be that chronic inflammation leads to both PD-L1 expression and malnutrition by transforming growth factor β, which is an immunosuppressive cytokine and abundant in the TME of MPM^41^ and implicates the promotion of cancer cachexia^42^. In the present study, the PD-L1 IC score was inversely correlated with PNI, but the PD-L1 IC score exhibited no relationship with non-epithelioid histology, which could be due to malnutrition-induced immune dysfunction^43^, and the cause of malnutrition could be chronic inflammation. In contrast, a positive correlation between PD-L1 IC and NLR or PLR might result in either PD-L1-induced inactivation of circulating lymphocytes or chronic inflammation-induced increase in the number of both neutrophils and platelets. Moreover, PNI was inversely correlated with platelet count, CRP, NLR, C/NLR and PLR, whereas it was positively correlated with LMR, suggesting that tumor-induced chronic inflammation and malnutrition coexist in patients with MPM.

Survival impact of these five parameters was determined using the log-rank test and univariate analysis, which demonstrated that NLR, PNI, and C/NLR were significant prognostic factors for patients with MPM who underwent EPP, and multivariate analysis indicated that NLR was an independent prognostic factor for 5-year OS in patients with MPM who underwent EPP. Our findings suggest that NLR test, one of the least invasive and cheapest blood tests, could predict the clinical outcome of EPP; moreover, PD-L1 status in TME does independently exhibit any impact on the outcome despite the cost, time, and effort required for its evaluation. A previous study on patients with MPM reported a high NLR (NLR > 3.0) at baseline as a poor prognostic factor in MPM treated with or without chemotherapy^9^. Another study reported that NLR > 3.0 was an independent prognostic marker associated with shorter survival for 85 patients who underwent EPP, which is in accordance with our results^10^. In contrast, Tagawa et al. reported that PLR < 215 was associated with improved survival in patients with MPM who underwent EPP, whereas NLR was not identified as a prognostic marker in their study^44^. Our results did not demonstrate a significant impact of PLR on the prognosis of MPM patients who underwent EPP, although a low PLR (< 167.1) predicted good prognosis. Yamagishi et al. reported that among NLR, PLR, and LMR, only LMR was an independent predictor of OS in patients with MPM, although patients with both operative and inoperative MPM were included in the study^45^. Therefore, it is unclear whether NLR is a definitive prognostic marker for OS, and further studies are required to establish whether inflammatory and nutritional markers such as NLR and PLR are reliable predictive markers for EPP. The combination of NLR and PNI may also be a promising parameter because in a previous study, low NLR with high PNI was reported to be a good prognostic factor for resectable hepatocellular carcinoma^46^, but our results demonstrate only low NLR as a good prognosis factor.

A limitation of this study is the small sample size; however, it is difficult to obtain data for a large number of patients with MPM who underwent EPP because of the rarity of this condition. The strength of this study is that to the best of our knowledge, this is the first study to evaluate five inflammatory/nutritional parameters simultaneously and to assess their relationship with PD-L1 expression in the TME of patients with MPM who underwent EPP.

Thus, we demonstrated that NLR is an independent prognostic factor for patients with MPM who underwent EPP. Preoperative assessment of NLR could predict whether the prognosis of patients with MPM can be improved by EPP, which is an invasive surgery.

## Supplementary Information

Supplementary Information 1.

Supplementary Information 2.

Supplementary Information 3.

## Data Availability

The datasets generated during and/or analyzed during the current study are available from the corresponding author on reasonable request.

## Abbreviations

MPM: Malignant pleural mesothelioma
PD1: Programmed cell death protein 1
PD-L1: Programmed cell death protein 1 ligand-1
NLR: Neutrophil to lymphocyte ratio
PNI: Prognostic nutritional index
CRP: C-reactive protein
EPP: Extrapleural pneumonectomy
TME: Tumor microenvironment
C/NLR: C-reactive protein/NLR ratio
PLR: Platelet/lymphocyte ratio
LMR: Lymphocyte/monocyte ratio
MCR: Macroscopic complete resection
IHC: Immunohistochemistry
OS: Overall survival
pStage: Pathological stage
WBC: White blood cell count
PD-L1 TC: PD-L1 by tumor cells
PD-L1 IC: PD-L1 by immune cells
ROC: Receiver operating characteristics
Rs: Spearman’s rank correlation coefficient.
HR: Hazard ratio
CI: Confidence interval
MDSC: Myeloid-derived suppressor cells
AUC: Area under the curve
MST: Median survival time
