# Supplementary material for: Preoperative neutrophil-to-lymphocyte ratio correlates with PD-L1 expression in immune cells of patients with malignant pleural mesothelioma and predicts prognosis
Source: Sci Rep. 2023 Mar 31;13:5263. doi: 10.1038/s41598-023-31448-4 (PMC10066199; doi:10.1038/s41598-023-31448-4)
Supplement: Supplementary file 1 — Supplementary Information 1. [file 41598_2023_31448_MOESM1_ESM.docx]

**Supplementary Figure Legends**

**Supplementary Figure 1**

Receiver operating characteristic (ROC) curves of single inflammatory or immunological parameters for predicting 5-year overall survival. ROC curves for (**a**) WBC, (**b**) platelet count, (**c**) PD-L1 TC score, and (**d**) PD-L1 IC are shown. WBC, white blood cell count; AUC, area under the curve; CI, confidence interval; PD-L1, programmed cell death 1 ligand-1, TC, by tumor cells; IC, by immune cells. **p*<0.05.

**Supplementary Figure 2**

Receiver operating characteristic (ROC) curves different combinations of inflammatory and nutritional parameters for predicting 5-year overall survival. ROC curves for (**a**) NLR, (**b**) PNI, (**c**) C/NLR, (**d**) PLR, and (e) LMR are shown. NLR, neutrophil-to-lymphocyte ratio; PNI, prognostic nutritional index; C/NLR, C-reactive protein-to-NLR ratio; PLR, platelet-to-lymphocyte ratio; LMR, lymphocyte-to-monocyte ratio; AUC, area under the curve; CI, confidence interval. **p*<0.05.
