# Supplementary figures and images for: Preoperative neutrophil-to-lymphocyte ratio correlates with PD-L1 expression in immune cells of patients with malignant pleural mesothelioma and predicts prognosis
Source: Sci Rep. 2023 Mar 31;13:5263. doi: 10.1038/s41598-023-31448-4 (PMC10066199; doi:10.1038/s41598-023-31448-4)

# Supplemental Figure 1

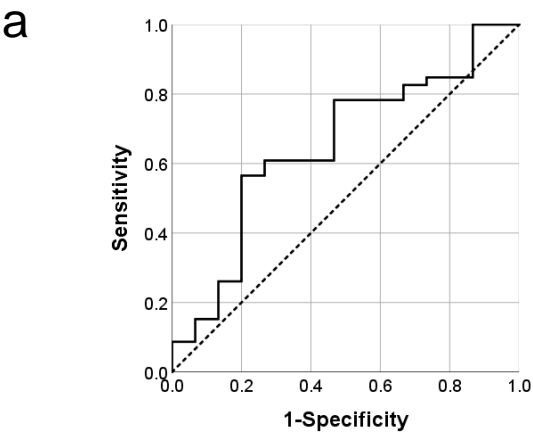

WBC > 5965  
AUC 0.651,  $p=0.082$   
95%CI 0.488-0.813

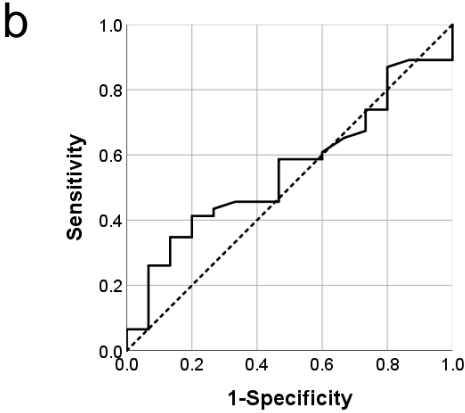

Platelet count > 28.35  
AUC 0.554,  $p=0.530$   
95%CI 0.400-0.709

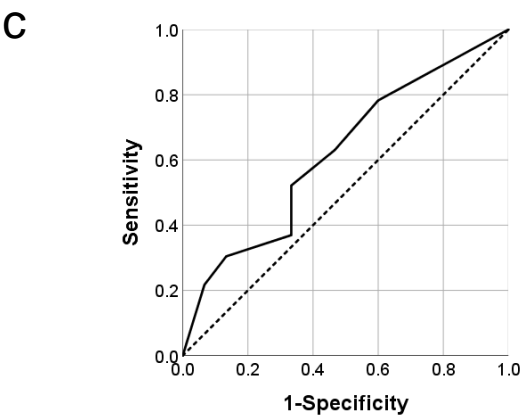

PD-L1 TC > 1.75  
AUC 0.620,  $p=0.167$   
95%CI 0.457-0.782

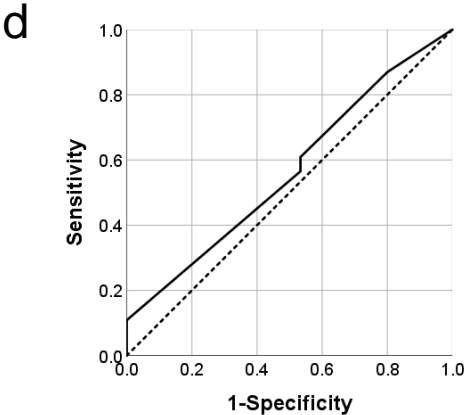

PD-L1 IC > 1.75  
AUC 0.564,  $p=0.461$   
95%CI 0.400-0.728

Supplement: Supplementary file 2 — Supplementary Information 2. [file 41598_2023_31448_MOESM2_ESM.pdf]

# Supplemental Figure 2

a

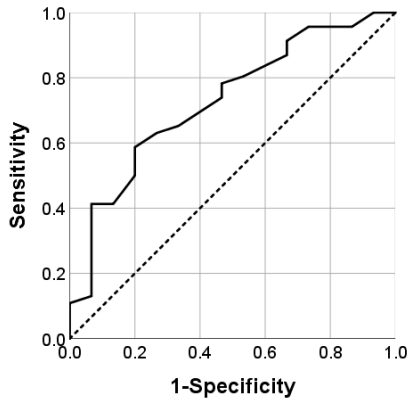

NLR > 2.850  
AUC 0.728,  **$p=0.008^*$**   
95%CI 0.583-0.873

b

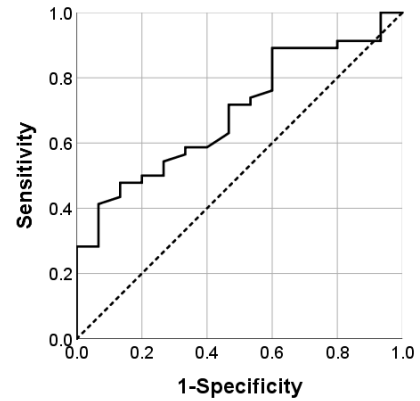

PNI < 45.150  
AUC 0.693,  **$p=0.025^*$**   
95%CI 0.553-0.834

c

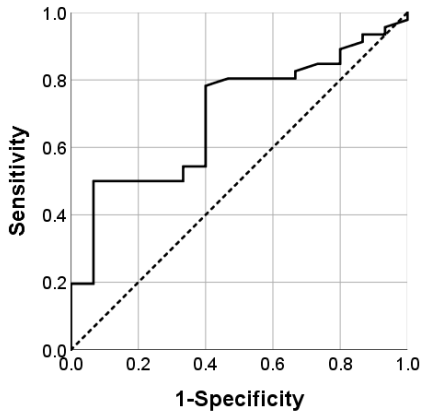

C/NLR > 0.955  
AUC 0.696,  **$p=0.024^*$**   
95%CI 0.554-0.838

d

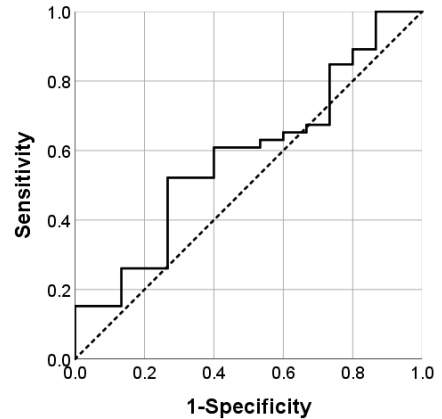

PLR > 167.1  
AUC 0.586,  $p=0.323$   
95%CI 0.421-0.750

e

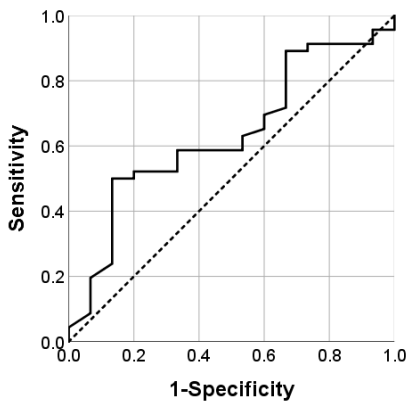

LMR < 5.15  
AUC 0.635,  $p=0.119$   
95%CI 0.478-0.792

Supplement: Supplementary file 3 — Supplementary Information 3. [file 41598_2023_31448_MOESM3_ESM.pdf]
